# Brain herniation in a patient with apparently normal intracranial pressure: a case report

**DOI:** 10.1186/1752-1947-4-297

**Published:** 2010-08-31

**Authors:** Mats B Dahlqvist, Robert H Andres, Andreas Raabe, Stephan M Jakob, Jukka Takala, Martin W Dünser

**Affiliations:** 1 Department of Intensive Care Medicine, Bern University Hospital and University of Bern, Bern, Switzerland; 2 Department of Neurosurgery, Bern University Hospital and University of Bern, Bern, Switzerland

## Abstract

**Introduction:**

Intracranial pressure monitoring is commonly implemented in patients with neurologic injury and at high risk of developing intracranial hypertension, to detect changes in intracranial pressure in a timely manner. This enables early and potentially life-saving treatment of intracranial hypertension.

**Case presentation:**

An intraparenchymal pressure probe was placed in the hemisphere contralateral to a large basal ganglia hemorrhage in a 75-year-old Caucasian man who was mechanically ventilated and sedated because of depressed consciousness. Intracranial pressures were continuously recorded and never exceeded 17 mmHg. After sedation had been stopped, our patient showed clinical signs of transtentorial brain herniation, despite apparently normal intracranial pressures (less than 10 mmHg). Computed tomography revealed that the size of the intracerebral hematoma had increased together with significant unilateral brain edema and transtentorial herniation. The contralateral hemisphere where the intraparenchymal pressure probe was placed appeared normal. Our patient underwent emergency decompressive craniotomy and was tracheotomized early, but did not completely recover.

**Conclusions:**

Intraparenchymal pressure probes placed in the hemisphere contralateral to an intracerebral hematoma may dramatically underestimate intracranial pressure despite apparently normal values, even in the case of transtentorial brain herniation.

## Introduction

Elevated supratentorial intracranial pressure (ICP) can cause transtentorial brain herniation, leading to cerebral hypoperfusion, brainstem herniation, and ultimately death, if left untreated [1]. ICP monitoring is, therefore, commonly implemented in patients with neurologic injury and a high risk of developing intracranial hypertension, in order to detect changes in ICP in a timely manner and to induce therapeutic interventions [2]. Measurement of ICP appears particularly important in patients who cannot be clinically evaluated due to sedation [2].

In this case report, we present a patient who experienced transtentorial brain herniation despite an apparently normal ICP.

## Case presentation

**Figure 1:**
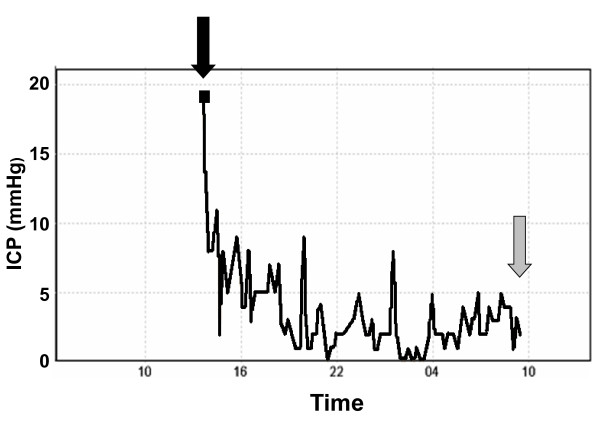
**Continuously recorded intracranial pressure readings from the time of insertion of the intraparenchymal pressure probe (black arrow) to the time that clinically evident brain herniation appeared (grey arrow)**.

**Figure 2:**
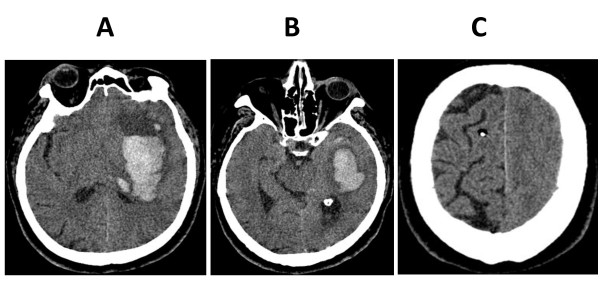
**CT scan images after clinical signs of brain herniation developed**. (A) Basal ganglia hemorrhage with perifocal edema. (B) Left-sided transtentorial herniation of the parahippocampal gyrus. (C) Edema of the left hemisphere with normally appearing right hemisphere.

A 75-year-old Caucasian man with chronic arterial hypertension, hyperlipidemia and chronic obstructive pulmonary disease presented to the emergency department with right-sided hemiplegia and facial nerve paresis, global aphasia and gaze deviation to the left. His level of consciousness was depressed (Glasgow Coma Scale 11) and arterial blood pressure was elevated (200/90 mmHg). There was no history of trauma or known coagulation disorder. An urgently performed magnetic resonance imaging (MRI) examination revealed a large left-sided basal ganglia hemorrhage. After admission to the intensive care unit, our patient's level of consciousness further deteriorated (Glasgow Coma Scale 6) and he had to be intubated to protect his airway. Since he could not be clinically evaluated due to sedation required for endotracheal tube tolerance and mechanical ventilation, ICP monitoring was indicated. An intraparenchymal pressure probe (Spiegelberg PN; Spiegelberg GmbH, Hamburg, Germany) was uneventfully inserted into the right hemisphere. The ICP measured was initially 17 mmHg, but rapidly decreased with adequate sedation using a continuous propofol infusion and repeated fentanyl injections. By that time, arterial blood pressure had decreased to tolerable levels (150/90 mmHg) and heart rate was moderately reduced to 50 to 60 bpm after endotracheal intubation. After several hours, during which the ICP remained low (Figure 1) and the arterial blood pressure and heart rate remained stable, propofol infusion was stopped to clinically evaluate our patient. Soon afterwards our patient started to show extensor posturing in reaction to pain, with sinus bradycardia (heart rate 30 to 35 bpm) and severe arterial hypertension (systolic arterial blood pressure more than 220 to 240 mmHg) rapidly developing. Pupils were mid-sized and pupillary responses were maintained. Paradoxically, during the development of typical clinical signs of transtentorial brain herniation, ICP remained low and never exceeded 10 mmHg (Figure 1). Our patient was immediately rushed to the radiology department where computed tomography (CT) scanning detected an increase in both hemorrhage and perifocal edema size (Figure 2A). This mass lesion caused ipsilateral displacement of the parahippocampal gyrus into the tentorial notch (Figure 2B), as well as subfalxial brain herniation. Whereas the left hemisphere was edematous with loss of gyral differentiation, the interhemispheric falx was not displaced, and the right hemisphere, where the intraparenchymal catheter was placed, appeared structurally normal (Figure 2C). In an emergency surgical procedure, decompressive craniotomy was performed. Post-operatively, right-sided hemiplegia persisted and the level of consciousness remained depressed. Therefore, early tracheotomy on intensive care unit day three was performed. Ten days following the intracerebral hemorrhage, our patient was discharged from the intensive care unit. When he entered long-term neurologic rehabilitation another ten days later, his neurologic function of our patient remained impaired (right-sided hemiparesis, Glasgow Coma Scale 10 to 11, no communication possible).

## Discussion

Traditionally, intraparenchymal pressure probes are placed in one of the frontal lobes in an attempt to minimize complications [3]. Although some study results have been controversial [4], a notable amount of evidence indicates that marked and clinically relevant ICP gradients within the supratentorial compartment may exist in patients with neurologic injury [3,5-7]. ICP gradients of up to 28 mmHg were reported in humans [8]. In contrast to diffuse lesions, interhemispheric ICP gradients were detected in almost half of patients with a focal brain lesion. In most of these cases, ICP was higher in the vicinity of the focal mass and correlated directly with its size [3,5-7].

The guidelines in our center require that ICP be recorded at the site of the lesion. However, the guidelines were not followed in this case. In our patient, the ICP gradient was so high that "normal" ICP was measured in the right hemisphere while transtentorial herniation of the left hemisphere occurred. The size and location of intracerebral hemorrhage could explain why even a moderate increase in left-hemispheric ICP may have caused transtentorial brain herniation in our patient and did not increase right-hemispheric ICP first. Thirty years ago, Papo *et al. *suggested that neurological deterioration and even brain herniation may occur in the absence of significant ICP changes in patients with intracerebral hemorrhage [9]. Even though we cannot definitely exclude the possibility, it is unlikely that an artifact of the ICP monitor caused the observed discrepancy of brain herniation in our patient with apparently low ICP. CT images showing a massively swollen left hemisphere with a structurally normal right hemisphere underline this assumption. Furthermore, substantial drifts of intraparenchymal pressure devices usually do not occur immediately after insertion [10].

Although this report describes a single patient, we believe that relevant lessons for ICP measurement in patients with intracerebral hemorrhage can be drawn from this case. First, placement of an intraparenchymal probe into the hemisphere contralateral to a large intracerebral hemorrhage may grossly underestimate ICP around the hematoma. This can be particularly devastating when hemorrhage occurs in deep brain structures such as the basal ganglia which are anatomically close to the tentorium. Considering experimental study results [11,12], it must be assumed that placement of the intraparenchymal pressure probe on the ipsilateral side of the lesion would have allowed detection of locally elevated ICP in our patient. Furthermore, one can hypothesize that measurement of ICP through an intraventricular sonde might have rendered even more reliable ICP results than placement of a left-sided intraparenchymal sonde in the presented patient. Since intraventricular sondes record the pressure of the cerebrospinal fluid which distributes equally throughout the supratentorial compartment, it is unlikely that they are prone to recording false low ICPs even if relevant supratentorial ICP gradients are present.

## Conclusions

Intraparenchymal pressure probes placed in the hemisphere contralateral to an intracerebral hematoma may dramatically underestimate ICP and render apparently normal values even in the case of transtentorial brain herniation.

## Abbreviations

CT: computed tomography; ICP: intracranial pressure; MRT: magnetic resonance tomography.

## Competing interests

The authors declare that they have no competing interests.

## Authors' contributions

MBD made substantial contributions to conception of this case report, acquired and interpreted data, drafted the manuscript and gave final approval of the version to be published. RA acquired and interpreted data, critically revised the manuscript for important intellectual content and gave final approval of the version to be published. AR acquired and interpreted data, critically revised the manuscript for important intellectual content and gave final approval of the version to be published. SJ interpreted data, critically revised the manuscript for important intellectual content and gave final approval of the version to be published. JT interpreted data, critically revised the manuscript for important intellectual content and gave final approval of the version to be published. MWD made substantial contributions to conception of this case report, acquired and interpreted data, drafted the manuscript and gave final approval of the version to be published.

## Consent

Written informed consent for publication of this case report and any accompanying images was obtained from the patient's next of kin. A copy of the written consent is available for review by the Editor-in-Chief of this journal.
